# Dramatic down-regulation of oxidoreductases in human hepatocellular carcinoma hepG2 cells: proteomics and gene ontology unveiling new frontiers in cancer enzymology

**DOI:** 10.1186/1477-5956-6-29

**Published:** 2008-10-24

**Authors:** Lambert CM Ngoka

**Affiliations:** 1 Department of Chemistry, Virginia Commonwealth University, 1001 West Main Street, P. O. Box 842006, Richmond, VA 23284-2006, USA; 2 Department of Biomedical Sciences, Paul L. Foster School of Medicine, Texas Tech University Health Sciences Center, MSB-1, USA

## Abstract

**Background:**

Oxidoreductases are enzymes that catalyze many redox reactions in normal and neoplastic cells. Their actions include catalysis of the transformation of free, neutral oxygen gas into oxygen free radicals, superoxide, hydroperoxide, singlet oxygen and hydrogen peroxide. These activated forms of oxygen contribute to oxidative stress that modifies lipids, proteins, DNA and carbohydrates. On the other hand, oxidoreductases constitute one of the most important free radical scavenger systems typified by catalase, superoxide dismutase and glutathione peroxidase.

In this work, proteomics, Gene Ontology mapping and Directed Acyclic Graphs (DAG) are employed to detect and quantify differential oxidoreductase enzyme expressions between HepG2 cells and normal human liver tissues.

**Results:**

For the set of bioinformatics calculations whose BLAST searches are performed using the BLAST program **BLASTP 2.2.13 [Nov-27-2005]**, DAG of the Gene Ontology's Molecular Function annotations show that oxidoreductase activity parent node of the liver proteome contains 331 annotated protein sequences, 7 child nodes and an annotation score of 188.9, whereas that of HepG2 cells has 188 annotated protein sequences, 3 child nodes and an annotation score of only 91.9. Overwhelming preponderance of oxidoreductases in the liver is additionally supported by the isomerase DAGs: nearly all the reactions described in the normal liver isomerase DAG are oxidoreductase isomerization reactions, whereas only one of the three child nodes in the HepG2 isomerase DAG is oxidoreductase. Upon normalization of the annotation scores to the parent Molecular Function nodes, oxidoreductases are down-regulated in HepG2 cells by 58%.

Similarly, for the set of bioinformatics calculations whose BLAST searches are carried out using **BLASTP 2.2.15 [Oct-15-2006**], oxidoreductases are down-regulated in HepG2 cells by 56%.

**Conclusion:**

Proteomics and Gene Ontology reveal, for the first time, differential enzyme activities between HepG2 cells and normal human liver tissues, which may be a promising new prognostic marker of Hepatocellular carcinoma.

Two independent sets of bioinformatics calculations that employ two BLAST program versions, and searched different databases, arrived at essentially the same conclusion: oxidoreductases are down-regulated in HepG2 cells by approximately 57%, when compared to normal human liver tissues. Down-regulation of oxidoreductases in hepatoma is additionally supported by Gene Ontology analysis of isomerises.

## Background

Hepatocellular carcinoma (HCC or hepatoma) is the most common primary cancer of the liver [1]. It is the fifth most common cancer worldwide with about one million new diagnoses annually [1]. The seventh most common cause of cancer deaths in men, and the ninth in women [2], HCC accounts for nearly 80–90% of all liver cancers [3]. It has been shown that more than 80% of individuals with HCC have cirrhosis [4,5], and that hepatitis B virus (HBV) [6], hepatitis C virus (HCV) [6] and aflatoxin B1 (AFB) [6] account for up to 80% of all HCCs [7]. To date, the most widely recognized biomarker of HCC is alpha-fetoprotein, which is elevated in the blood of nearly 70% of patients diagnosed with this disease [8].

A distinctive pathological hallmark of Hepatocellular carcinoma is a dramatic down-regulation of oxidoreductase enzymes (oxidoreductases) in the host, when compared to matched healthy cohorts [9-27]. The genetic and biochemical determinants underlying this phenomenon are not known. Additionally, many structural and functional abnormalities in oxidoreductases have been linked to Hepatocellular carcinoma [9-27].

Oxidoreductase enzymes are key enzymes in pathways of oxygen utilization in normal and neoplastic cells. Their actions include the conversion of molecular oxygen to oxygen free radicals, superoxide, hydroperoxide, singlet oxygen and hydrogen peroxide. These activated forms of oxygen contribute to oxidative stress that modifies lipids, proteins, DNA and carbohydrates. Oxidoreductases also constitute the most important free radical scavenger systems exemplified by catalase, superoxide dismutase and glutathione peroxidise [19].

Repression of oxidoreductases in hepatoma has been consistently documented in humans, animal models and cell lines [9-27]. In one study, several oxidoreductase enzymes, including cytochrome oxidase, succinate dehydrogenase, monoamine oxidase, urate oxidase, D-amino acid oxidase, L-α-hydroxy acid oxidase, xanthine oxidase and catalase, were examined; the enzyme activities of all the oxidoreductase are steeply reduced in hepatoma, when compared to controls [15]. Other work [18-24,27,28] show that, in Hepatocellular carcinoma, the natural free radical scavenger systems of oxidoreductase enzymes that protect cells from oxidative stress, apoptosis and other damaging effects of oxygen free radicals, are strongly compromised. Sierra-Rivera and co-workers [22] noted that the decline in enzymatic activities of CuZnSOD, MnSOD and catalase in hepatoma was due to a decline in the levels of immunoreactive proteins. Another study [11] found that cytochrome oxidase was about 60% lower in whole-cell suspensions of Morris hepatoma 3924A than in whole-cell suspensions of normal or host rat liver. Weber and co-workers [13,14] observed that xanthine oxidase, the key rate-limiting enzyme of purine catabolism, was decreased 2- to 10-fold in all hepatomas studied, regardless of the degree of malignancy, growth rates and degrees of the histological differentiation of the neoplasms.

A wide range of enzyme assays and other experimental methods have been employed to study oxidoreductase enzymes in Hepatocellular carcinoma. They include: reverse transcriptase polymerase chain reaction amplification [25,26], immunohistochemical staining [25,26,28], *in-situ *hybridization [25], and Western blotting [26,28]. Many oxidoreductase enzyme assays incorporate spectroscopic absorbance [11,13,15,28] and polarography [11]. Other utilize RNA blot hybridization [21], run-on assays [21] and Lowry protein assays [11,15].

Although the above experimental methods have contributed immensely to better understanding of the pathobiology of oxidoreductase enzymes in hepatic neoplasia, they all suffer from lack of specificity in the structural information they provide (for example, specific posttranslational modifications of proteins), ability to analyze sample molecules in the presence of interfering contaminants and ability to map the broad cellular biology and biophysical profiles of the tumor vs. matched benign cohorts.

To date, no proteomics method for oxidoreductase enzymes in Hepatocellular carcinoma has been attempted or documented. The mass spectrometry-based proteomic approach presented in this work holds the potential to overcome all of the above limitations, in addition to providing improved ease of automation, speed and sensitivity.

HepG2 cell line, rather than hepatoma, is chosen for proteomic comparison with normal human liver in this work. The reason for choosing a cell line is because heterogeneity inherently associated with complex liver tumor matrix, which could be further compounded by cirrhosis, hepatitis B virus, Hepatitis C virus, inflammation, regenerative liver fibrosis and other lesions, may introduce inordinate errors.

Unique challenges posed by the heterogeneity of complex liver tumor matrix is attested by the work of Fernandez and co-workers [29], who clearly showed that the variation within adenocarcinoma tissue samples is considerably greater than that within the matched benign cohorts. Therefore, HepG2 cell line is chosen for this study primarily because it is more homogenous. However, tumor cell lines do not always accurately represent the *in vivo *biological profiles of the tumor tissues from which they are derived. For example, Sandberg and co-workers [30] found that only 34 of the 60 cell lines used in a quantitative tissue similarity index analysis were most similar to the tumor types from which they were derived. In a study [31], freshly dissected non-cultured HLE cells from both central and peripheral lens epithelia were found to exhibit different protein expression patterns compared to corresponding immortalized HLE B-3 cell line.

Sometimes, however, the protein expression profiles of cell lines do represent the accurate protein expression profiles of the *in vivo *tumor or tissues. Indeed, immortalized cell lines derived from human tumors, like HepG2 cell line, are more homogenous than solid liver tumors, as noted above. They have been extensively employed in a wide range of *in vitro *disease models, and they produce large amounts of high quality DNA, mRNA and protein for analysis. Furthermore, they are excellent tools for mechanistic studies and are commercially available. Additionally, they afford improved reproducibility, ease of application of quantitative techniques and controlled experimental conditions. In addition, the protein profiles of cell cultures tend to give cleaner backgrounds than those of the corresponding tissues.

Wirth and co-workers [32] provide an example where a cell line reflects a true representation of the *in vivo *biology: they reported that proteins present in non-transformed cell lines Chang and WRL-68 were identical to the proteins found in normal human liver. And, in a proteome profiling study, fresh bladder tumors showed strikingly similar protein expression profiles when compared to their primary cell cultures [33]. Furthermore, Didonato and co-workers[34] compared the protein expressions of renal cell carcinoma (RCC) tissues and patient-matched normal kidney tissues with the primary RCC renal cell cultures derived from the tissues, and found that the overall patterns of the profiles of the tissues and cell cultures were similar. The protein spots on the 2D-PAGE of the cell cultures had much higher resolution, and the exact expression alterations of proteins in cancer and normal cell cultures were present in the tissue samples. Tan and co-workers [35] examined an immortalized mouse retinal cell line (661W) for markers characteristic of photoreceptor cells; they found that the 661W cell line exhibited similar cellular and biochemical characteristics to those of the original cone photoreceptor cells from which they were derived. In a T-cell acute lymphoblastic leukemia study, Gjerset and co-workers [36] found that, with regard to surface markers, karyotype, and T-cell receptor gene rearrangements, the cell cultures indeed closely resembled those of the patients at the time of diagnosis.

Comparative proteomics of a normal tissue against those of its tumor cell line could afford useful insights into qualitative and quantitative changes in proteins profiles following tumor development, and facilitate the discovery of novel markers of tumors. Loredana and co-workers [37] profiled the proteome of normal human breast tissues, and compared these with those of the corresponding 8701-BC breast cancer cell line (ductal infiltrating carcinoma, DIC). They found that the 8701-BC cell line retains the dominant luminal phenotype of the breast epithelium, which expresses predominantly cytokeratins -8 and -18, and that the proteomic profile of the cell line appears highly homologous to the *in vivo *counterpart. Emili and co-workers [38] performed a comparative proteomics of protein expression patterns in proliferating MCF-7 breast cancer cells and normal human mammary epithelial cells using gel-free shotgun proteomics. They observed important differences in the levels of key regulators of the cell cycle, signal transduction, apoptosis, transcriptional regulation, and cell metabolism.

The benefits of using cancer cell lines in cancer research are clearly demonstrated by the **In Vitro Cell Line Screening Project **(IVCLSP) at the National Cancer Institute's Developmental Therapeutics Program . The IVCLSP screens up to 3,000 drug compounds every year for potential anticancer activity, using 60 different human tumor cell lines, representing leukemia, melanoma and cancers of the lung, colon, brain, ovary, breast, prostate, and kidney. The success of this program can only mean that cell lines do provide valuable biological data, and sometimes, accurate representation of the *in vivo *biology of tumors.

The present study was designed to employ multidimensional protein identification technology (MudPIT) proteomics, Gene Ontology mapping and directed acyclic graph representations to detect, compare and contrast qualitative and quantitative oxidoreductase enzyme expression profiles of the HepG2 proteome vs. that of a normal human liver.

Two independent sets of bioinformatics calculations that employ two BLAST program versions, and searched different sets of databases, arrived at essentially the same conclusion – that oxidoreductases are down-regulated in HepG2 cells by an average of 57%, when compared to normal human liver tissues.

## Methods

### Samples

HepG2 cell lysates (RDI-HepG2-CPX Lot#HEPG2/3b), 500 μg in 0.5 mL (1 mg/mL) in SDS-PAGE Buffer (62 mM Tris pH6.8, 2% SDS, 0.9% β-mercaptoethanol, 0.003% bromophenol blue, 5% glycerol), and normal human liver tissue lysates (RDI-NLH-01 Lot #NLH01/082604a), 150 μg in 0.032 mL (5 μg/μL) denaturing buffer with proteolytic inhibitors to minimize proteolytic damage to proteins, all lysates extracted by the method of Laemmli [39], were obtained from RDI Division of Fitzgerald Industries Intl (Concord, MA) and stored at -80°C until use. Prior to use, the lysates are aliquotted into 100 μg total protein portions and stored at -80°C. A 100 μg aliquot is used for each experiment.

Under the auspices of Virginia Commonwealth University Office of Research Subjects Protection, compliance with U.S. Department of Health and Human Services (Office for Human Research Protection) regulations at 45 CFR 46.101(b)(4) was provided by Fitzgerald Industries.

### 2D cleanup

Proteins are separated from buffers, detergents, salts and other contaminants using a 2D clean-up kit and protocol provided by Amersham Biosciences (GE Healthcare, Piscataway, NJ). The kit consists of four reagents: a precipitant that pellets the proteins, a co-precipitant that enhances the removal of the proteins from the solution, a wash buffer that removes non-protein contaminants from the protein precipitate, and a wash additive that promotes rapid and complete re-suspension of the proteins.

Prior to the beginning of clean-up, the wash buffer was chilled at -20°C for 1 hr. After thawing and spinning down 100 μg aliquots of HepG2 cell lysates and normal human liver tissue lysates, 300 μL precipitant was added. The mixture was vortexed on Eppendorf Thermomixer R (Eppendorf North America, Westbury, NY), and then incubated in ice for 15 minutes. Next, 300 μL of co-precipitant was added and the mixture mixed. The mixture was centrifuged at 12000 × g for 5 minutes to pellet the proteins. The clear supernatant liquid was carefully pipetted out while retaining the protein precipitate at the bottom of the 1.5 mL Eppendorf tube. Without disturbing the pellet layer, 40 μL of co-precipitant was added to the top of the pellet; the mixture was kept in ice for 5 minutes before centrifuging it again at 12000 × g for another 5 minutes. The pellet was dispersed by adding 25 μL of MilliQ water and centrifuging for 10 minutes. After adding 1 mL of chilled wash buffer at -20°C and 5 μL of wash additive, the mixture was vortexed once every 30 seconds for a total of 35 minutes. At this point, the proteins did not dissolve, but dispersed. The mixture was again centrifuged at 12000 × g for 5 minutes. The supernatant was carefully discarded, and the pellet dried. The pellets are amorphous.

### In-solution digestion

The dried pellet was re-suspended in 20 μL 8 M urea/100 mM ammonium bicarbonate (ABC), and 0.6 μL of 100 mM Dithiothreitol (DTT) in 100 mM ABC (i.e. 3 mM DTT) was stirred in Eppendorf Thermomixer R for 1 hr at 29°C. After adjusting to room temperature, 1.5 μL of 200 mM iodo-acetamide (IAA) in 100 mM ABC (final concentration of 15 mM IAA) was added. Alkylation was then carried out by incubating the mixture for 45 minutes in a darkroom. Then 1.5 μL of 200 mM DTT/100 mM ABC was added to consume any un-reacted IAA. The urea concentration was reduced to about 1 M by diluting the mixture with 140 μL of (50 mM ABC+2 mM CaCl_2_). Digestion was carried out by adding 6 μL of 0.40 μg/μL = 2.4 μg of Promega Sequencing Grade trypsin and incubating in Eppendorf Thermomixer R for 20 hr at 37.4°C. At the end of the 20-hour incubation, the reaction was stopped by adding 4.0 μL of 2% acetonitrile, and 6 μL of 10% TFA was added to adjust the pH to 5.0.

### Desalting

Manual, Micro Trap desalting cartridge and protocol from Michrom (Michrom BioResources, Auburn, CA) were used. First, the microTrap was washed with 80 μL of LC/MS Solvent B (90%ACN/0.1% TFA). Next, it is equilibrated with 80 μL of LCMS Solvent A (2%ACN/0.1% TFA). Then, 20 μL of peptide digest sample is loaded onto the microTrap; salts are removed by washing with 50 μL aliquots of LCMS solvent A (2%ACN/0.1% TFA). Tryptic peptides are eluted from the micro Trap with 16 μL of 70% ACN. Desalted peptides are evaporated to dryness on an SC2 SpeedVAC^® ^Plus Thermo savant (Thermo Fisher Scientific, Waltham, MA).

#### Nanospray

The nanospray is a Paradigm Nanotrap Platform equipped with a Paradigm Metal spray needle. The spray tip is a 7.5 cm long, 30 μm (Internal Diameter) × 105 μm (Outer Diameter) surgical stainless steel, electrochemically cut and polished, and sheathed by a 125 μm PEEK Tubing. The needle is electrochemically cut and polished. It permits flow range of 0.5 to 10 μL/min, and a voltage range of 1000 to 5000 Volts. A 1/16" stainless steel Valco nut attaches the spray needle to a 1/16" to 1/16" Valco union, which is mounted on the Nanotrap platform.

### Nanospray source parameters

Sheath Gas Flow Rate = 0; Aux Gas Flow Rate = 0; Spray Voltage (kC) = 2.51; Spray Current (μA) = -0.05; Capillary Temp (°C) = 221.10; Capillary Voltage (V) = 9.22; Tube Lens Offset (V) = 50.

#### Multidimensional nano-HPLC

The nano-HPLC is a Paradigm MS4B Multi-Dimensional HPLC equipped with a Michrom Paradigm AS1 refrigerated autosampler and XCalibur software plugin (Michrom BioResources, Auburn, CA). It is configured and operated in a 3-1 column-switching arrangement. Pump D is used for sample loading on captrap cartridge (sample concentration and de-salting) at 50 μL/minute for 5 minutes.

#### Columns (Michrom BioResources)

• Peptide Nanotrap (TR5/25109/42): 150 μm × 50 mm; 400 nL volume.

• Nanotrap analytical column (CL5/61241/00): 5 μm 200 Ǻ Magic C_18 _75 μm × 150 mm.

• SCX Captrap (TR1/25108/35): Contains a medium pore, large particle, silica-based strong cation exchange material (PolySulfoethyl Aspartamide). Binds protein digests, peptides, and other molecules (0.5–50 kD) for 1D or 2D analysis; concentrates samples up to 100 fold (pH range 2.7–7.0).

### Nano-LC/ESI-MS/MS

One-dimensional (1D) and two-dimensional (2D) nano HPLC experiments are run at a flow rate of 300 nL/min. Samples are loaded onto trap columns for concentration and desalting at 50 μL/min. For each experiment, 12 μL of peptide digest resulting from 100 μg total protein is used: 2 μL is injected for 1D; 10 μL for 2D. Each shotgun experiment consists of a 12-cycle MudPIT run in which a 60-minute nano-LC gradient is run for each of: 1D, 2D, 2D (0 mM NH4COO), 2D (25 mM NH4COO), 2D (50 mM NH4COO), 2D (75 mM NH4COO), 2D (100 mM NH4COO), 2D (150 mM NH4COO), 2D (200 mM NH4COO), 2D (250 mM NH4COO), 2D (300 mM NH4COO) and 2D (500 mM NH4COO).

#### Mass spectrometry

Data-dependent MS and MS/MS spectra are acquired on an LCQ Deca Xp plus (Thermo Fisher Scientific, San Jose, CA).

### MS and MS/MS

Five scan events are recorded for each data acquisition cycle. The first scan event is used for full scan MS acquisition from 300–1800 *m/z*. Data are recorded in the **centriod **mode only because the first scan event does not permit **profile **mode for data acquisition. The remaining four scan events are used for collisionally activated dissociation: the four most abundant ions in each MS are selected and fragmented to produce product ion mass spectra. All tandem spectra are recorded in the **profile **mode.

### MS/MS parameters

Number of microscans: 4; Maximum Injection Time (ms): 200; Isolation width (*m/z*): 3; Normalized Collision Energy (%): 35; Activation Q: 0.250; Activation Time (msec): 30.00; Scan Range: 300–1800 *m/z*.

#### Database searches and protein identification

Proteins are identified by searching the MS/MS spectra against NCBI nr human fasta, using Bioworks v3.2 (Thermo Fisher Scientific, San Jose, CA). The **Unified Search Results File **format (*.SRF) is employed, rather than the traditional SEQUEST *.DTA and *.OUT formats. Peptide and protein hits are scored and ranked using the new probability-based scoring algorithm and the new Final Score (Sf) that are incorporated in Bioworks 3.2. Because MS/MS spectra are acquired in the **profile **mode, each Nano-LC/ESI-MS/MS run range in size from 95 – 120 Megabytes, depending on the number of microscans (typically 4 ms) in the Advanced Define Scan function in the MS/MS LCQtune file of the **Instrument Method**.

### Filters

Only peptides identified as possessing fully tryptic termini (containing up to two missed internal trypsin cleavage sites), with cross-correlation scores (X_corr_) greater than 1.9 for singly charged peptides, 2.3 for doubly charged peptides and 3.75 for triply charged peptides, are used for peptide identification. In addition, the delta-correlation scores (ΔC_n_) must be greater than 0.1 for peptide identification.

With protein probability set at ≤ 1e-002, the 77831 original hits (i.e. using the default filter settings) returned by Bioworks 3.2 on the HepG2 proteome are reduced to 1795 validated, unique proteins (Additional File 1). Similarly, 1819 validated, unique proteins (Additional File 2) are obtained from 79961 original hits of the normal human liver proteome.

### Gene ontology

Gene ontology (Michael Ashburner [40,41]) analyses are carried out with Blast2GO [42]; a java webstart-enabled Gene Ontology annotation, visualization and analysis program. The 1024 MB option of Blast2GO was installed. The calculations, as implemented here, consist of three key sequential steps: (a) Basic Local Alignment Search Tool (BLAST) [43,44], (b) Mapping and (c) Annotation.

All calculations are carried out on "Medusa," a high performance dual tower, 64-node Beowulf cluster having 32 Gigabytes of SDRAM and 1 Terabyte of storage. Medusa is located at the Bioinformatics Computational Core Laboratory at Virginia Commonwealth University's Center for the Study of Biological Complexity.

**a) BLAST: **In BLAST [43], protein input queries are submitted to the BLAST server at the National Center for Biotechnology Information (NCBI) of the National Institutes of Health (NIH) over the internet . The BLAST server generates hits (hit gene ids (gi) and gene names/accessions; 40 hits for each query; eValue Cutoff = 0.001) needed for the Mapping step (below). The BLAST server accepts only ***fasta***-formatted protein sequences as input query.

The 1795 HepG2 proteins and the 1819 normal human liver proteins are converted into **fasta **formats and submitted to NIH-NCBI BLAST server for calculations. The BLAST server generates a Blast Table for each of the 1795 HepG2 and 1819 normal human liver proteins. The Blast Table contains the results of the calculations: Sequences producing significant alignments, Gene Name, ACCESION #, e-Value, align-length, positives, similarity %, hsps, mapping and UniProt. These results are then input to the Mapping algorithm (below).

**b) Mapping: **The mapping algorithm uses the parameters of the Blast Table to search various databases to identify and retrieve Gene Ontologies (GO) associated with the hits obtained from NCBI BLAST searches. The results of Mapping are presented in a Sequence Table, which consists of nine parameters: Sequence name, Seq description, Length, #hits, Maximum eValue, Similarity mean, #GOs found, #GO IDs, Enzyme (i.e. Enzyme Commission #).

**c) Annotation: **The annotation procedure selects the GO terms from the GO pool obtained by the Mapping step and assigning them to the query sequences, using Annotation Rule. Annotations are validated and expanded using an annotation expander. The expander deploys an additional Gene Ontology structure: the Second Gene Ontology layer, to suggest new Biological Processes and Cellular Components, based on the gene's existing Molecular Function annotations.

**Gene Ontology **(GO) is a consortium comprising some of the world's major animal, plant and microbial databases of genes and gene products, whose key objective was to provide a coherent, species-independent platform for accurate descriptions of gene products across different databases.

Central to the Gene Ontology project are three structured controlled vocabularies known as Ontologies. Ontologies describe gene products in terms of their associated Biological Processes (BP), Molecular Functions (MF) and Cellular Components (CC). A Biological Process is a set biochemical actions accomplished by one or more ordered assemblies of Molecular Functions, while Molecular Function itself is the specific, elemental task performed by individual gene products or assembled complexes of gene products. GO molecular function terms represent biological activities (not the molecular entities that perform the task; they do not specify the time or cellular site or in what context the action takes place. Cellular Component Ontology, as the name implies, describes the cellular sites, at the levels of sub-cellular structures and macromolecular complexes, where the gene product is found.

Results of a **Gene Ontology **analysis are presented in the form of Directed Acyclic Graph (DAG). A DAG is a hierarchical representation of Ontology terms in a way that depicts the directional relationships between parent-child GO term nodes. A DAG differs from a *simple hierarchy graph *in that a child (or more specialized term) may have more than one parent.

There are two types of relationships between the terms in parent-child child nodes of DAG. A child node that represents a more specific instance of a parent node is designated as ***'is a***' whereas ***'part of' ***denote a child term node that is a constituent of the parent node term. The ***'part of' ***is slightly more complicated than the ***'is a***' relationship. For example, A ***'part of' ***B means that whenever A is present, it is always a part of B, but A does not always have to be present. Example, nucleus is ***'part of' ***a cell (nuclei are always part of a cell) but not all cells have nuclei.

Every **Gene Ontology **annotation must provide valid evidence, known as **Evidence Codes **(EC), which was used to support it. Evidence codes encompass a broad range of empirical or other support such as electronic annotation or direct assay.

## Results

The HepG2 dataset (Additional File 3, A) consists of twelve MudPIT mass spectra (one **1D **Nano-LC/ESI-MS/MS and eleven **2D **Nano-LC/ESI-MS/MS). Similarly, the normal human liver dataset (Additional File 3, B) has twelve MudPIT mass spectra. All mass spectra, with the exception of two HepG2 chromatograms (obtained with 0.0 mM NH_4_COO^- ^and 25.0 mM NH_4_COO^-^, respectively), show strong chromatograms with reasonable elution profiles, good signal/noise ions and reproducibility.

**Figure 1:**
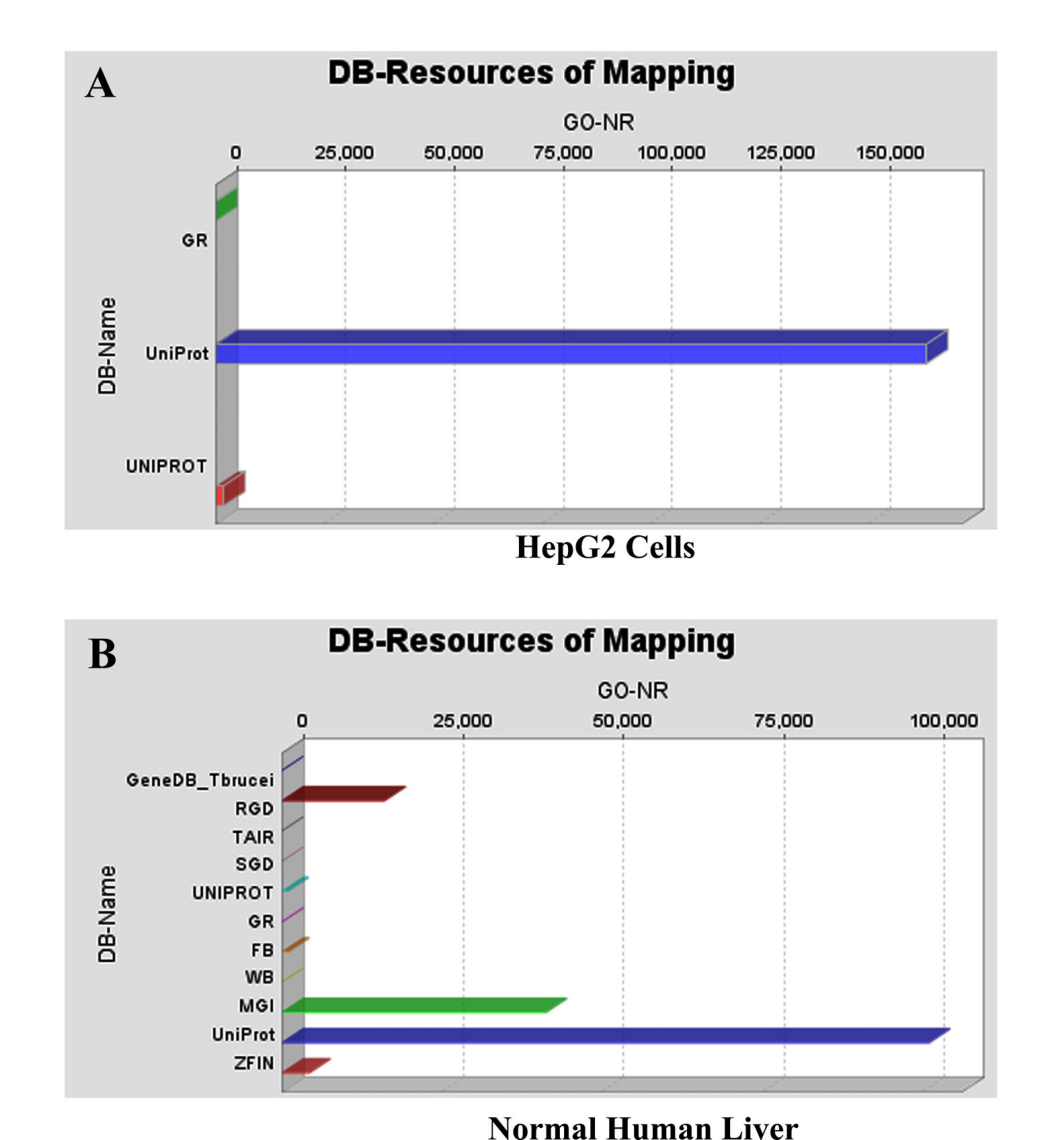
**DB-Resources of Mapping, for HepG2 (Panel A), and Normal Human Liver (Panel B), showing the databases where mapping found the Ontologies shown in Additional Files **4** and **5**, respectively.** BLAST searches were carried out with BLASTP 2.2.13 [Nov-27-2005].

As discussed in the **Methods **Section above, **Mapping **uses parameters of the **Blast Table **to search and retrieve Ontologies from various databases. The results are presented in a **Sequence Table**. The Sequence Tables for HepG2 and Normal Human Liver proteomes, for the set of analyses using the Blast program BLASTP 2.2.13 [Nov-27-2005], are shown in Additional Files 4 and 5, for HepG2 and Normal Human Liver, respectively. Mapping also returns DB-Resources of Mapping, shown here in Figure 1, for HepG2 (**Panel A**) and Normal Human Liver (**Panel B**), respectively.

**Figure 2:**
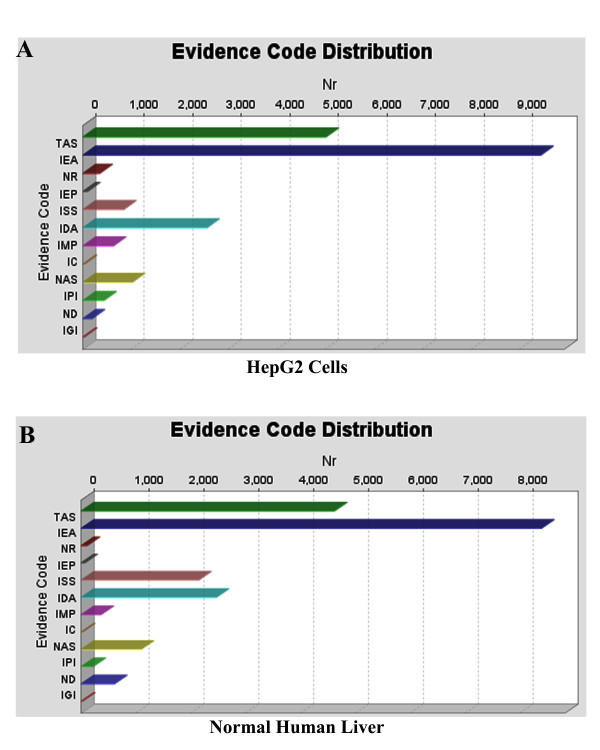
**Distribution of evidence codes that are found by Mapping, for the HepG2 and normal human liver proteomes, respectively.** These are for the set of bioinformatics analyses whose BLAST searches were carried out using BLASTP 2.2.13 [Nov-27-2005]. IC: Inferred by Curator, IDA: Inferred from Direct Assay, IEA: Inferred from Electronic Annotation, IEP: Inferred from Expression Pattern, IGC: Inferred from Genomic Context, IGI: Inferred from Genetic Interaction, IMP: Inferred from Mutant Phenotype, IPI: Inferred from Physical Interaction, ISS: Inferred from Sequence or Structural Similarity, NAS: Non-traceable Author Statement, ND: No biological Data available, TAS: Traceable Author Statement, NR: Not Recorded.

In the HepG2 proteome, (Figure 1A), gene Ontologies are found in three databases: GR, UniProt and UNIPROT, whereas in the normal human liver proteome (Figure 1B), Ontologies are found in eleven databases: GeneDB_Tbrucei, RGD, TAIR, SGD, UNIPROT, GR, FB, WB, MGI, UniProt and ZFIN. In addition, HepG2 proteome contains more Ontologies than normal human liver proteome. Figure 2 shows the distribution of the evidence codes for the HepG2 (**Panel A**) and normal human liver (**Panel B**) proteomes. The distributions are clearly characterized by diversity in EC, and dominated by traceable author statement (TAS), inferred from electronic annotation (IEA), and inferred from direct assay (IDA); these being the topmost ranks in the hierarchy of evidence codes [45].

**Figure 3:**
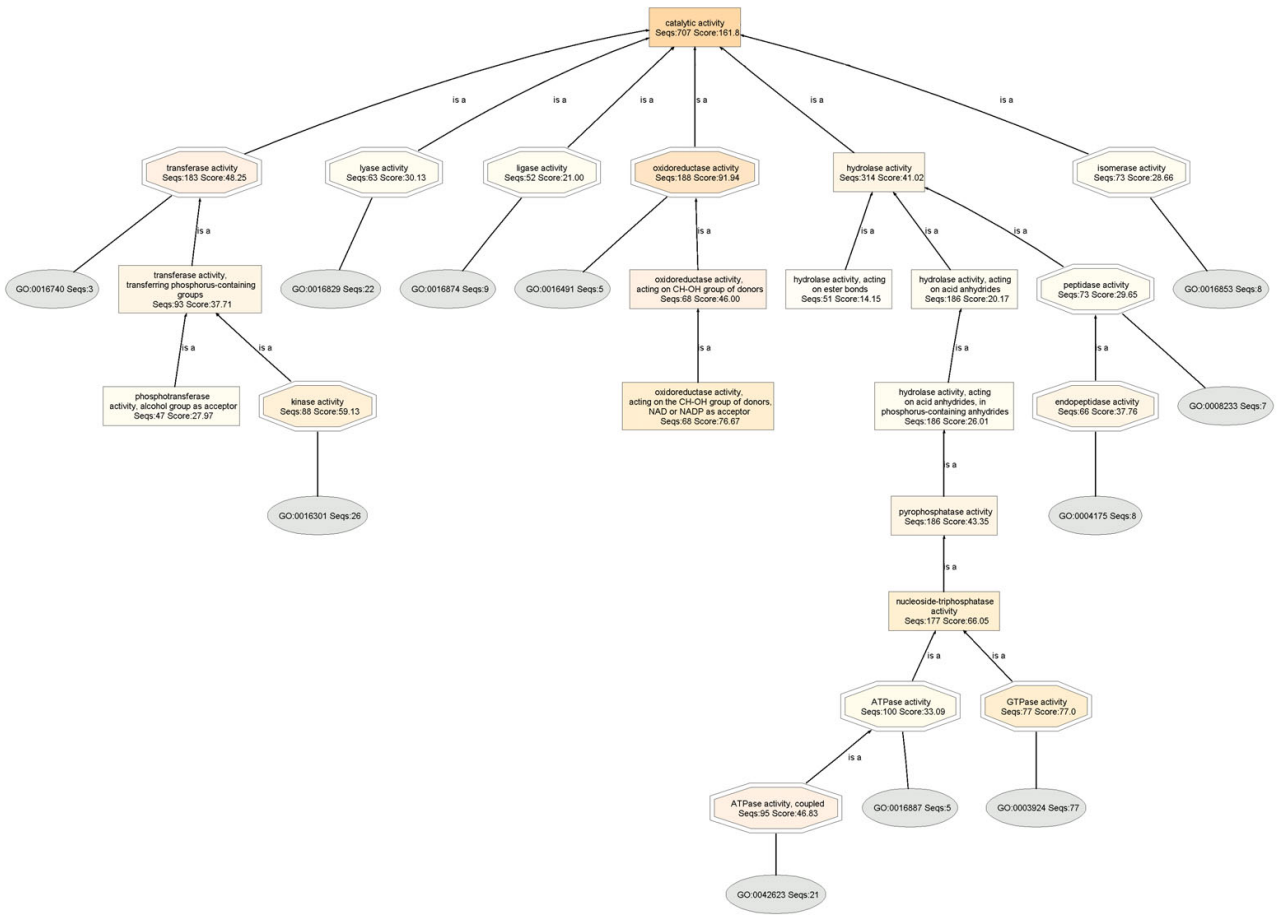
**Catalytic activity DAG of the HepG2 proteome**. BLAST searches were carried out with BLASTP 2.2.13 [Nov-27-2005].

In Figure 3, the catalytic activity DAG of the HepG2 proteome (Seqs:707 Score:161.8) has six level 2 child nodes corresponding to the six main classes of enzymes: transferase (Seqs:183 Score:48.25), lyase (Seqs:63 Score:30.13), ligase (Seqs:52 Score:21.00), oxidoreductase (Seqs:188 Score:91.94), hydrolase (Seqs:314 Score:41.02) and isomerases (Seqs:73 Score:28.66).

**Figure 4:**
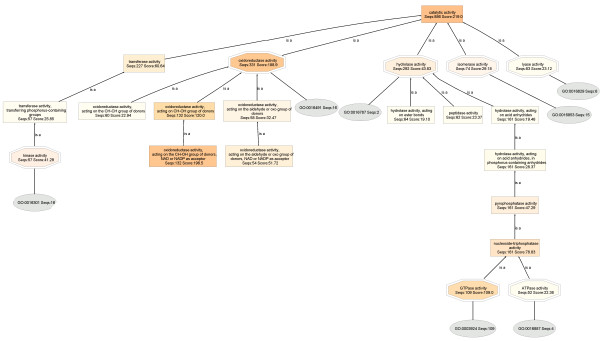
**Catalytic activity DAG of the normal human liver proteome**. BLAST searches were carried out with BLASTP 2.2.13 [Nov-27-2005].

On the other hand, the catalytic activity DAG of the normal human liver proteome (Seqs:886 Score:219.0) (Figure 4), has five child nodes: transferase (Seqs:227 Score:60.64), oxidoreductase (Seqs:331 Score:188.9), hydrolase (Seqs:292 Score:43.83), isomerases (Seqs:74 Score:29.18), and lyase (Seqs:83 Score:23.12).

On the basis of the resulting normalized annotation scores, it is seen that the overall catalytic activity is lower in HepG2 by 36%, despite the fact that more HepG2 proteins are annotated: HepG2: (Molecular Function: Seqs:1738 Score:1122.0; Catalytic activity, Seqs:707 Score:161.8) :: Liver: (Molecular Function: Seqs:1555 Score:967.1; Catalytic activity, Seqs:886 Score:219.0)].

**Figure 5:**
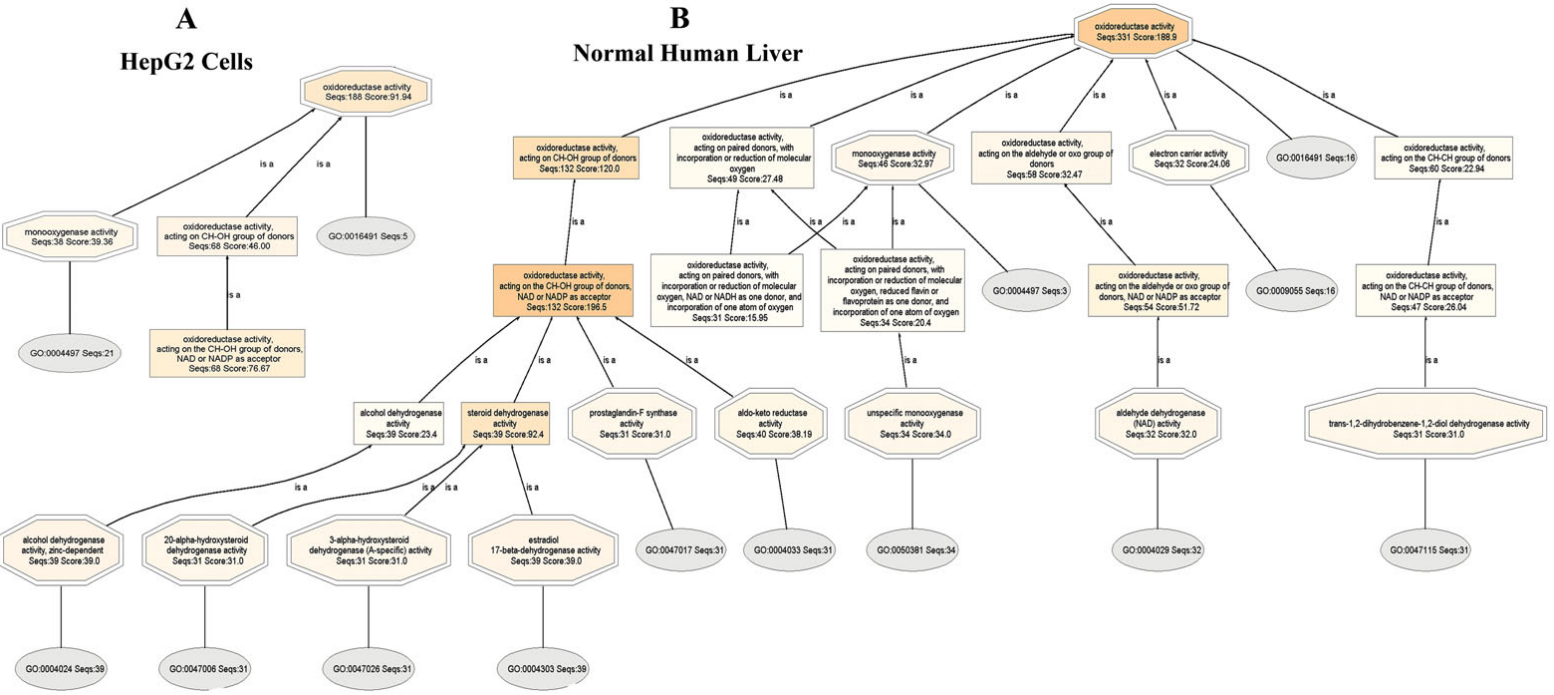
**Down-regulation of oxidoreductases in HepG2, when compared to normal human liver**. Oxidoreductase DAG for HepG2 (Panel A), and normal human liver (Panel B). Intramolecular oxidoreductase activity is down-regulated in HepG2, when compared with normal human liver. Node filter = 30, for both HepG2 and Normal Human Liver. BLAST searches were carried out with BLASTP 2.2.13 [Nov-27-2005].

Most importantly, normalized scores reveal that oxidoreductase enzymes are down-regulated in HepG2 cells by 58%: HepG2: (oxidoreductase activity, Seqs:188 Score:91.94) :: Liver: (oxidoreductase activity, Seqs:331 Score:188.9)]. Expanded views of the oxidoreductase DAGs are shown in Figure 5. Here, the node filter is fixed at 30 for both HepG2 and liver DAGs for an unbiased comparison.

A node filter is a means of simplifying the DAG: all nodes with 30 or lower protein sequences are not shown. It is seen that only three child nodes are observed for HepG2 oxidoreductase: monooxygenase activity (Seqs:38 Score:39.36), oxidoreductase activity, acting on CH-OH group of donors (Seqs:68 Score:46.00), and GO:0016491 (Seqs:5). The normal liver oxidoreductase, on the other hand, has seven child nodes, and whose sequences and scores are even greater: oxidoreductase activity, acting on CH-OH group of donors (Seqs:132 Score:120.0), oxidoreductase activity, acting on paired electron donors, with incorporation or reduction of molecular oxygen (Seqs:49 Score:27.48), monooxygenase activity(Seqs:46 Score:32.97), oxidoreductase activity, acting on the aldehyde or oxo group of donors (Seqs:58 Score:32.47), electron carrier activity (Seqs:32 Score:24.06), GO:0016491 (Seqs:16), and oxidoreductase activity, acting on CH-CH group of donors (Seqs:80 Score:22.94). In addition, a further lowering of the node filter on HepG2 oxidoreductase did not unveil additional child nodes that were previously cut off, whereas only a slight lowering of the node filter on normal liver oxidoreductase uncovers a large number of previously hidden child nodes, making the chart very complicated.

**Figure 6:**
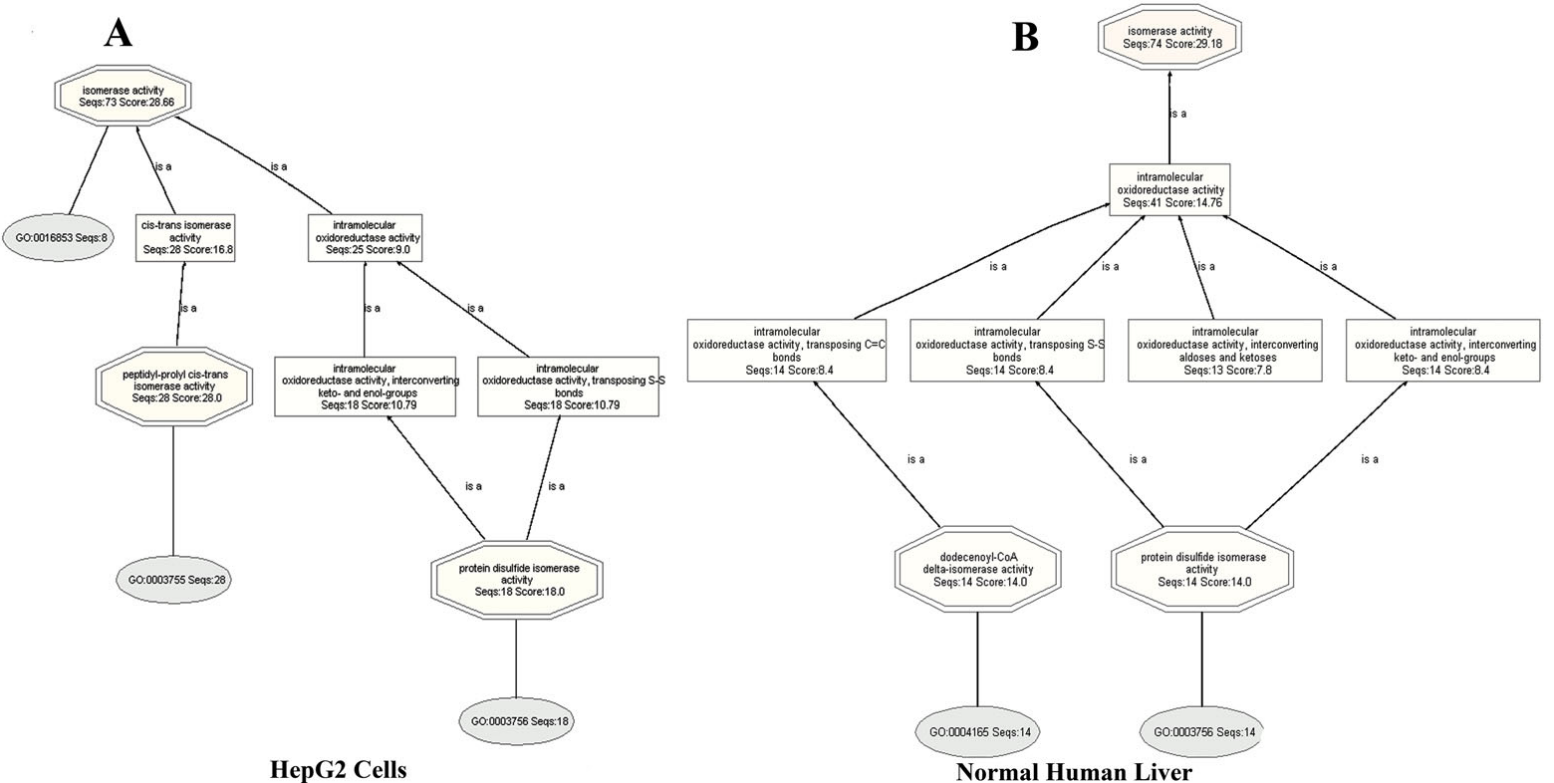
**Isomerase activity for HepG2 DAG (Panel A) and normal human liver proteomes (Panel B).** BLAST searches carried out with BLASTP 2.2.13 [Nov-27-2005].

Down-regulation of oxidoreductases in HepG2 cells is additionally supported by the isomerase DAGs (Figure 6), where intramolecular oxidoreductase activity is 39% higher in the normal liver: [HepG2: (intramolecular oxidoreductase activity, Seqs:25 Score:9.0) :: Liver: (intramolecular oxidoreductase activity, Seqs:41 Score:14.76)]. In addition, all the reactions described in the liver isomerase are oxidoreductase reactions, whereas only one of the three child nodes in the HepG2 isomerase DAG is oxidoreductase.

These findings can only mean that oxidoreductase enzymes are down-regulated in HepG2 cells, when compared to normal human liver. (cf. HepG2 contains even more annotated protein sequences than the normal liver, as discussed previously).

The percentages referred to in the above paragraphs are those of **normalized annotation scores**. Normalization allows for sequence-independent comparison of the HepG2 and normal Human liver annotations: catalytic activities are expressed as percentages of the total Molecular Function sequences and scores at the parent node (i.e. HepG2: (Molecular Function: Seqs:1738 Score:1122.0 :: Liver: (Molecular Function: Seqs:1555 Score:967.10))].

### Validation

An additional step was taken to verify the down-regulation of oxidoreductases in HepG2: the BLAST and Mapping experiments were repeated under a different set of experimental conditions.

**Figure 7:**
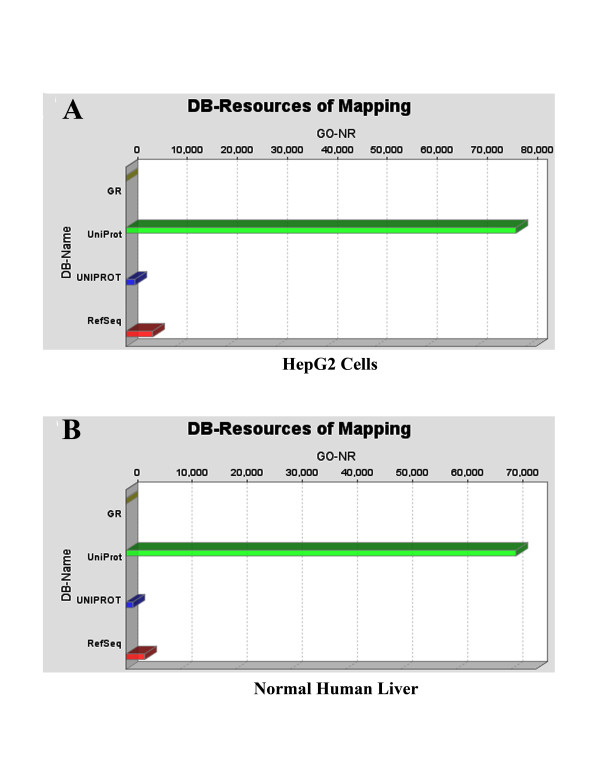
**DB-Resources of mapping, for HepG2 (Panel A), and Normal Human Liver (Panel B), showing the databases where mapping found the Ontologies that are presented in Additional Files **6** and **7**, respectively.** With BLASTP 2.2.15 [Oct-15-2006], mapping now includes RefSeq. Ontologies are found in four databases: GR, UniProt, UNIPROT and RefSeq. Ontologies found in this mapping are much less – almost half of the number found previously in Figure 1 (Panels A and B).

Here, a new BLAST program, **BLASTP 2.2.15 [Oct-15-2006] **was used. The preceding BLAST searches were carried out with **BLASTP 2.2.13 [Nov-27-2005]**, as discussed. The results from the BLAST calculations are used in the mapping step to search databases for Ontologies belonging to the BLAST hits. With **BLASTP 2.2.15 [Oct-15-2006]**, mapping now includes NCBI **RefSeq **database , and Ontologies are found in four databases: GR, UniProt, UNIPROT and RefSeq (Figure 7 (Panels **A **and **B**)); Ontologies found in this mapping are much less – essentially half of the number found previously in Figure 1 (Panels **A **and **B**).

**Figure 8:**
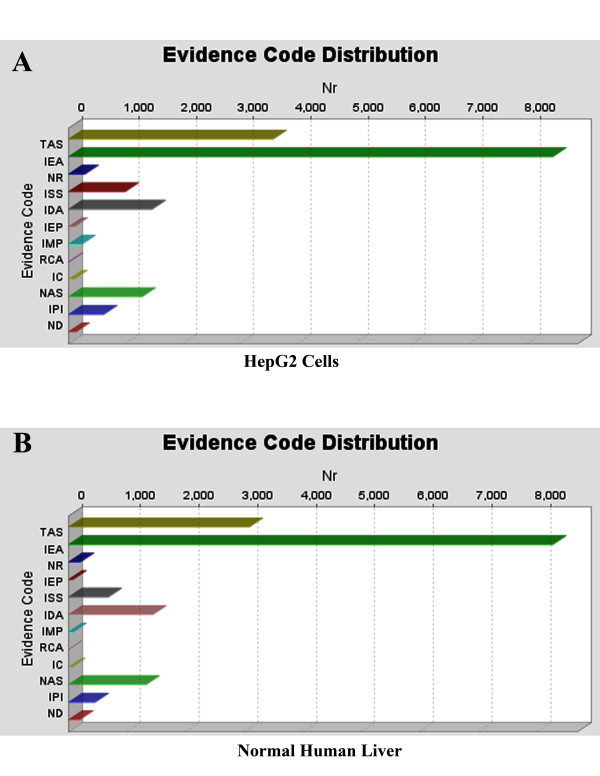
**Evidence code distribution, found by Mapping, for the HepG2 and normal human liver proteomes.** BLAST program, BLASTP 2.2.15 [Oct-15-2006] was used.

The distribution of evidence codes generated from the bioinformatics based on **BLASTP 2.2.15 [Oct-15-2006] **(Figure 8 (Panels **A **and **B**)) show very different profiles than those of **BLASTP 2.2.13 [Nov-27-2005] **of Figure 2.

The Sequence Tables, shown in Additional Files 6 and 7, for HepG2 and normal human Liver proteomes, respectively, are also vastly different from those of BLASTP 2.2.13 [Nov-27-2005].

**Figure 9:**
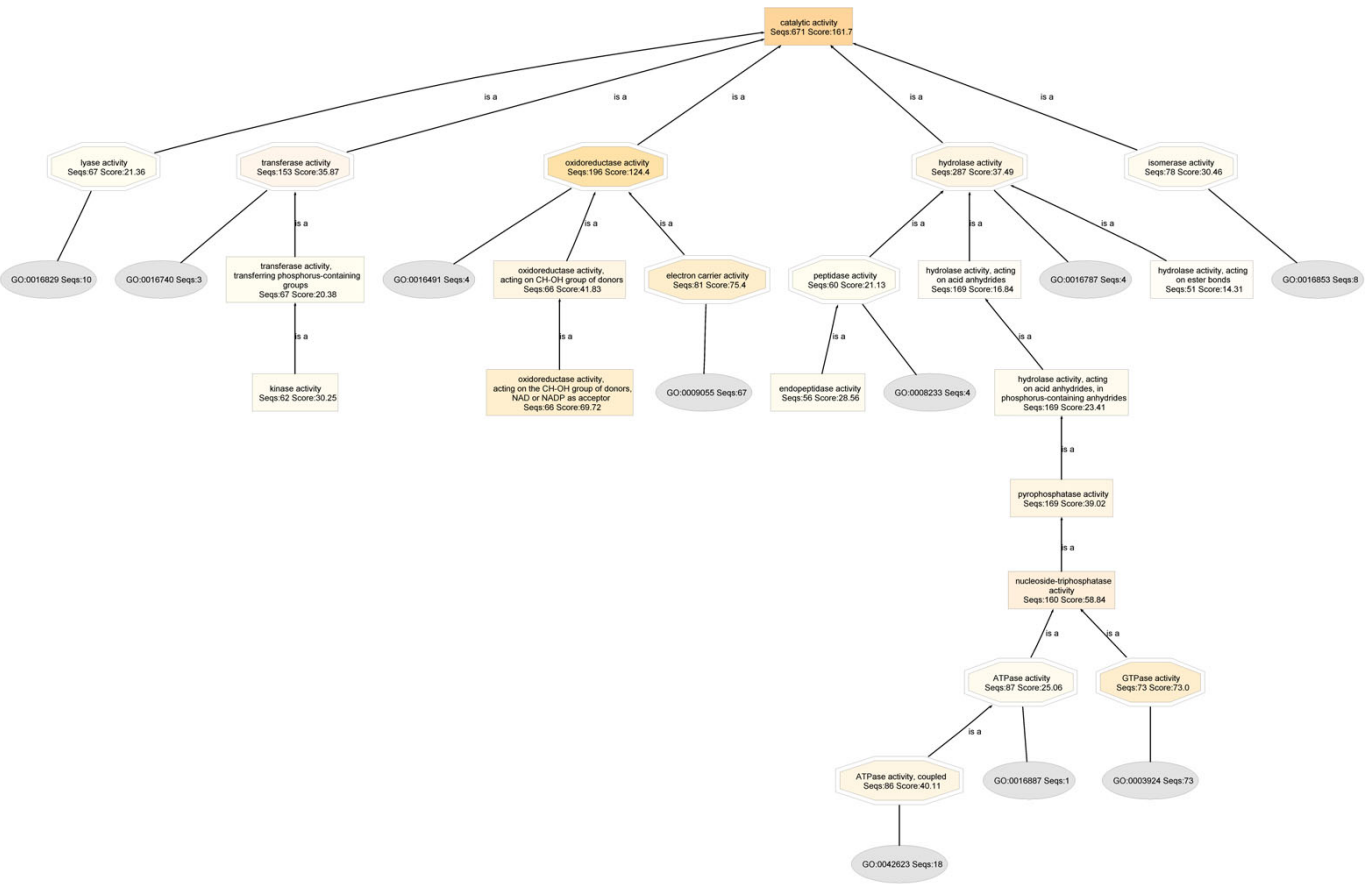
**Catalytic activity DAG of the HepG2 proteome.** BLAST searches were carried out with BLASTP 2.2.15 [Oct-15-2006].

The catalytic activity DAG of the HepG2 proteome (Seqs:671 Score:161.7) (figure 9) has five level 2 child nodes: transferase (Seqs:153 Score:35.87), lyase (Seqs:67 Score:21.36), oxidoreductase (Seqs:196 Score:124.4), hydrolase (Seqs:287 Score:37.49) and isomerase (Seqs:78 Score:30.46). Compared to those of BLASTP 2.2.13 [Nov-27-2005], the DAGs maintain comparatively similar profiles, Seqs and Scores.

**Figure 10:**
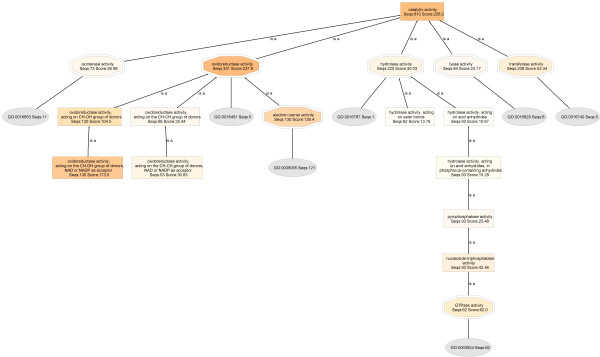
**Catalytic activity DAG of the normal human liver proteome.** BLAST searches were carried out with BLASTP 2.2.15 [Oct-15-2006].

In comparison, the catalytic activity DAG of the normal liver proteome (Seqs:810 Score:228.2) (Figure 10), also has five child nodes: transferase (Seqs:208 Score:53.34), oxidoreductase (Seqs:331 Score:237.8), hydrolase (Seqs:220 Score:30.03), isomerases (Seqs:73 Score:26.98), and lyase (Seqs:84 Score:23.17), but the profiles, Seqs and Scores are, in some cases, very different.

The catalytic activity is lower in HepG2 by 40%, despite the fact that more HepG2 proteins are annotated: HepG2: (Molecular Function: Seqs:1742 Score:953.1.0; Catalytic activity, Seqs:671 Score:161.7) :: Liver: (Molecular Function: Seqs:1559 Score:953.1; Catalytic activity, Seqs:810 Score:228.2)].

**Figure 11:**
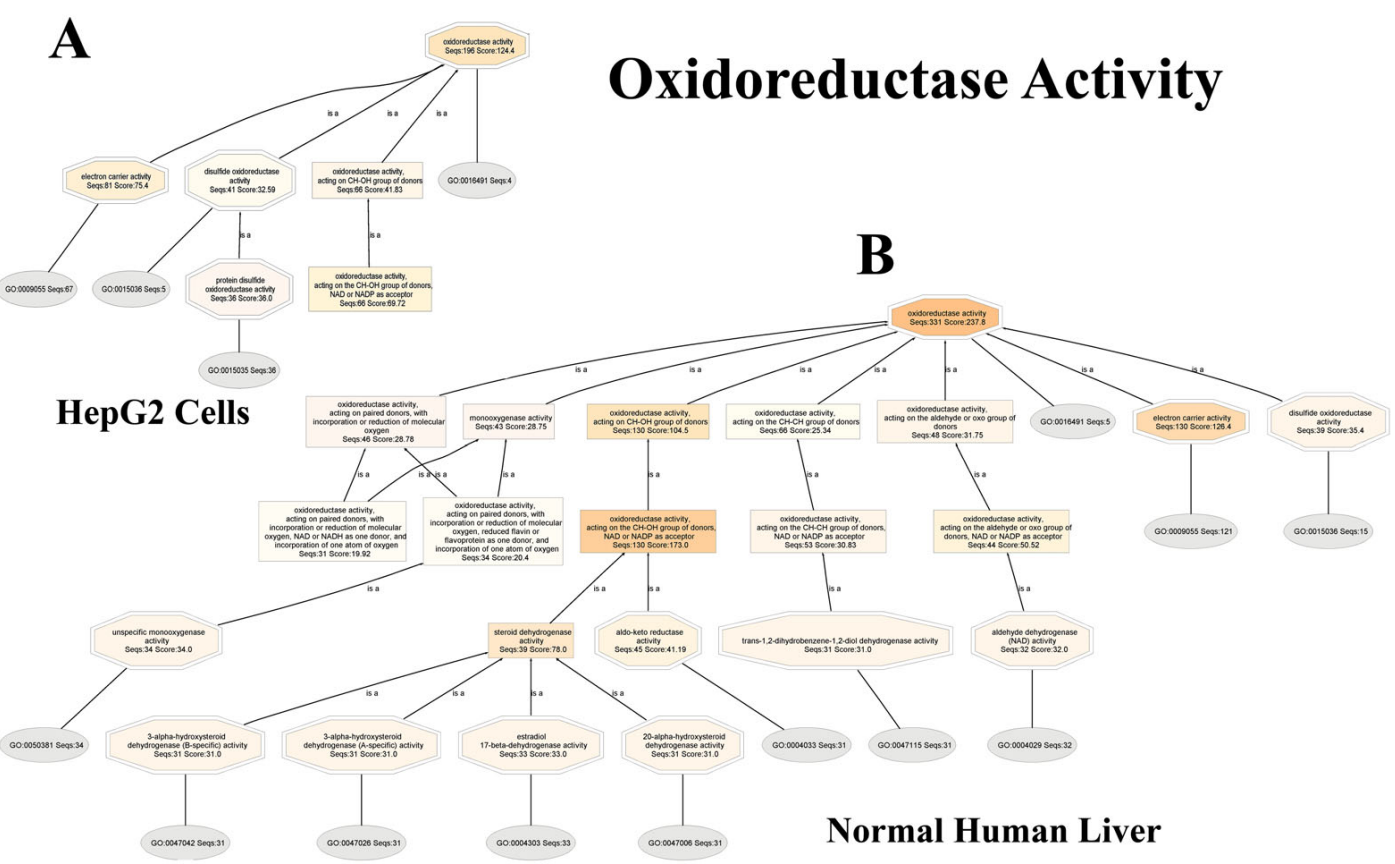
**Down-regulation of oxidoreductases in HepG2, when compared to normal human liver.** Oxidoreductase DAG for HepG2 (Panel A), and normal human liver (Panel B). Node filter = 30, for both HepG2 and Normal Human Liver. BLAST searches were carried out with BLASTP 2.2.15 [Oct-15-2006].

Based on normalized annotation scores, oxidoreductases are decreased in HepG2 by 56%: HepG2: (oxidoreductase activity, Seqs:196 Score:124.4) :: Liver: (oxidoreductase activity, Seqs:331 Score:237.8)]. Expanded views of the oxidoreductase DAGs are shown in Figure 11. Again, the node filter is fixed at 30 for both HepG2 and liver DAGs. It is seen that four child nodes are present in HepG2 oxidoreductase node: electron carrier activity (Seqs:81 Score:75.4), disulfide oxidoreductase activity (Seqs:41 Score:32.59), oxidoreductase activity, acting on CHOOH group of donors (Seqs:66 Score:41.83), and GO:0016491 (Seqs:4).

The normal liver oxidoreductase has eight child nodes: oxidoreductase activity, acting on CH-OH group of donors (Seqs:130 Score:104.5.0), oxidoreductase activity, acting on paired electron donors, with incorporation or reduction of molecular oxygen (Seqs:46 Score:28.78), monooxygenase activity (Seqs:43 Score:28.75), oxidoreductase activity, acting on the aldehyde or oxo group of donors (Seqs:48 Score:31.75), electron carrier activity (Seqs:130 Score:126.4), GO:0016491 (Seqs:5), disulfide oxidoreductase activity (Seqs:39 Score:35.4), and oxidoreductase activity, acting on CH-CH group of donors (Seqs:66 Score:25.25).

**Figure 12:**
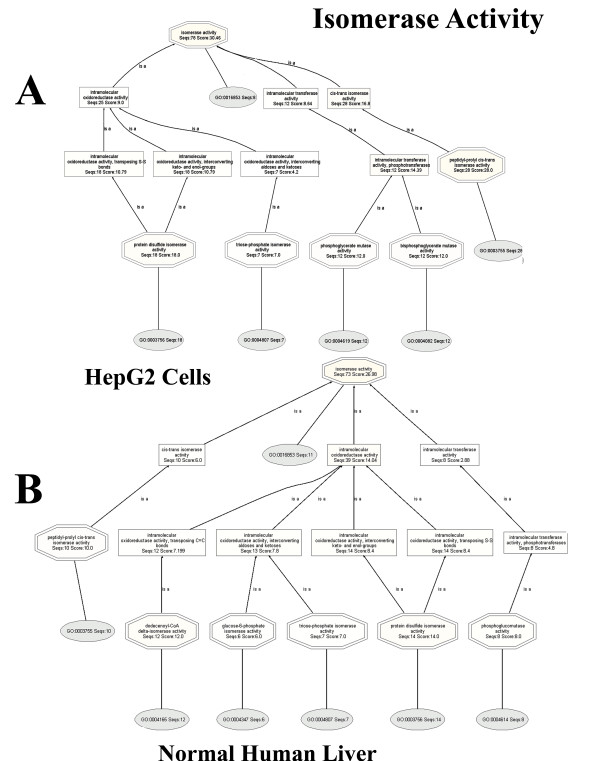
**Isomerase activity for HepG2 DAG (Panel A) and normal human liver proteomes (Panel B).** Intramolecular oxidoreductase activity is down-regulated in HepG2, when compared to normal human liver. BLAST searches were carried out with BLASTP 2.2.15 [Oct-15-2006].

Additional evidence in support of down-regulation of oxidoreductases in HepG2 cells are provided by intramolecular oxidoreductase activities, shown in Figure 12. The sequences and scores are remarkably similar to the values obtained with BLASTP 2.2.13 [Nov-27-2005] analyses of Figure 6: HepG2: (intra-molecular oxidoreductase activity, Seqs:25 Score:9.0) :: Liver: (intramolecular oxidoreductase activity, Seqs:39 Score:14.04)].

## Discussion

Oxidoreductase enzymes are extremely diverse in their structures, functions, cellular distribution and biochemical transformations they mediate. In fact, they are subdivided into 22 classes, based on the type of biochemical reaction pathway they catalyze [46]. One unique feature of oxidoreductases, however, is that they are strongly down-regulated in hepatic neoplasia. The exact reasons and/or molecular mechanisms are not known. Their fundamental roles as mediators of biochemical redox reactions may offer clues, however, although conclusive empirical evidence is scanty.

One possible clue may be found in the metabolic pathways of purine biosynthesis [47]. In fact, oxidoreductase enzymes that catabolize critical intracellular metabolites of *de novo *biosynthesis of purines are consistently documented to be highly down-regulated in hepatomas, the extent of which correlates with the degree and the severity of malignancy and tumor progression. At the same time, the enzymes that channel these strategic intermediates toward *de novo *biosynthesis of purine nucleotides and DNA/RNA are found to increase with increasing severity of the disease.

In non-neoplastic hepatocytes, the balance between these two opposing forces shifts in favor of greater catabolic capacity. In hepatic neoplasia, however, an apparent reprogramming of gene expression shifts the balance toward preponderance of anabolic capacity: a survival mechanism that confers selective advantage to cancer cells in an effort to maximize their proliferative potentials. Rapidly proliferating cancer cells have elevated demand for nucleic acid biosynthesis; they are critically dependent on abundant supply of key purine metabolites that are needed for the *de novo *biosynthesis of purine nucleotides adenine and guanine.

One such metabolite is inosine monophosphate (IMP), which is a precursor for *de novo *biosynthesis of purine nucleotides [14]. In hepatic neoplasia, all the key enzymes involved in the biosynthesis and utilization of IMP are found to increase [48-50], whereas the rate-limiting oxidoreductase enzyme of IMP catabolism, xanthine oxidase (EC:1.2.3.2), decreases sharply, in direct proportion to the severity of the disease [13,14].

**Figure 13:**
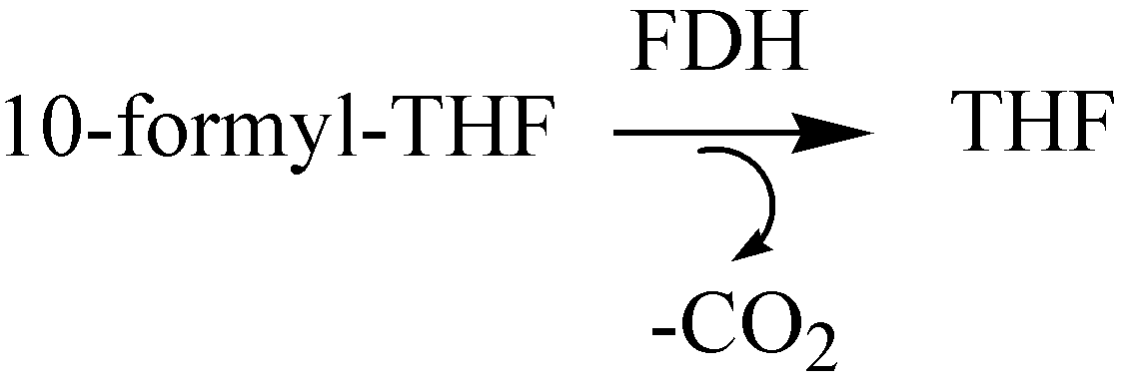
**_Oxidoreductase enzyme 10-formyltetrahydrofolate Dehydrogenase (FDH; EC:1.5.1.6), removes formyl group from 10-formyltetrahydrofolate (10-formyl-THF).** 10-formyl-THF is a critically needed precursor for two reactions of *de novo *biosynthesis of purine nucleotides.

Another example is 10-formyltetrahydrofolate (10-formyl-THF) [25,51]. 10-formyl-THF is a critically needed precursor for two reactions of *de novo *biosynthesis of purine nucleotides [52]. Its substrate is the oxidoreductase enzyme 10-formyltetrahydrofolate Dehydrogenase (FDH; EC:1.5.1.6), which removes its formyl group (Figure 13).

Thus, FDH plays a key role in the control of the intracellular 10-formyl-THF pool [51]. During malignant transformation and tumor progression, intracellular concentration of FDH is dramatically down-regulated by gene reprogramming [25,53], leading to a build-up of intracellular 10-formyl-THF pool. Concomitantly, the key enzymes of the *de novo *and salvage pathways of purine biosynthesis are increased [53].

Oxidoreductase scavenger enzyme systems of catalase (CAT), superoxide-dismutase (SOD), and glutathione peroxidase (GSH-Px) are the most important enzymatic free radical defense mechanisms that protect cells from apoptosis and other damaging effects oxidative stress. Research now show that CAT [19,21,54,55], SOD [23,24,56] and GSH-Px [16,54,56] are strongly down-regulated in Hepatocellular carcinoma, the putative explanation being likely, again, a genetic reprogramming in favor of high proliferative capacity of the cancer cells. Indeed, cancer cells evolve a variety of survival adaptations to boost their proliferative capacity.

Abundant supply of oxygen free radicals is highly needed by rapidly proliferating cancer cells [57]. And, cancer cells avoid apoptosis caused by excessive oxygen free radicals by activating protein kinase B (PKB; also known as c-Akt) which protects them from apoptosis [58].

Another possible explanation for the inhibition of CAT in hepatoma was suggested to stem from the secretion of a toxohormone from neoplastic tissue [59].

## Conclusion

Comparative proteomics and Gene Ontology analyses of the HepG2 Cells and normal human liver proteomes show that oxidoreductase enzymes are down-regulated in HepG2 cells by 57%. Specifically, when Gene Ontology Molecular Function annotations are plotted as Directed Acyclic Graphs, it is seen that the oxidoreductase activity parent node of the liver proteome consists of 331 protein sequences, 7 child nodes and an annotation score of 188.9, whereas that of HepG2 has 188 protein sequences, 3 child nodes and an annotation score of only 91.9.

Down-regulation of oxidoreductases in Hepatocellular carcinoma, when compared to surrounding non-neoplastic liver tissues, is well-documented in the literature, with examples that include 10-Formyltetrahydrofolate dehydrogenase, cytochrome oxidase, succinate dehydrogenase, monoamine oxidase, cytochrome P450, catalase, urate oxidase, D-amino acid oxidase, L-alpha-hydroxy acid oxidase, xanthine oxidase and (Cu/Zn) superoxide dismutase.

The exact reasons for this repression are not known, but one plausible explanation has been suggested to be an apparent reprogramming of gene expression, which shifts the metabolic balance toward preponderance of anabolic capacity over catabolism. This appears to be a survival mechanism which confers powerful selective advantage to cancer cells in an effort to maximize their proliferative potentials. Another possible explanation was suggested to stem from the secretion of a toxohormone by the cancer cells themselves (observed only in the case of inhibition of catalase in hepatoma).

This work is the first report on the use of proteomics, Gene Ontology and Directed Acyclic Graph representations to detect and quantify the repression of oxidoreductase enzymes in hepatoma. Detection of differential enzyme expressions between a tumor proteome and its non-neoplastic counterpart may offer the possibility of being a new prognostic marker for Hepatocellular carcinoma.

## Abbreviations

oxidoreductases: Oxidoreductase enzymes; MudPIT: multidimensional protein identification technology; DAG: Directed Acyclic Graph; GO: Gene Ontology; HCC: Hepatocellular carcinoma (hepatoma); BLAST: Basic Local Alignment Search Tool; NCBI: National Center for Biotechnology Information; *.SRF: Unified Search Results File; Sf: Final Score; CAT: catalase; SOD: superoxide dismutase; GSH-Px: glutathione peroxidase; PKB or c-Akt: protein kinase B; FDH: 10-formyltetrahydrofolate Dehydrogenase; THF: 10-formyltetrahydrofolate, 10-formyl; IMP: inosine monophosphate.

## Competing interests

The author declares that they have no competing interests.

## Authors' contributions

LCN carried out all of this work, using HepG2 cell lysates (RDI-HepG2-CPX Lot#HEPG2/3b) and normal human liver tissue lysates (RDI-NLH-01 Lot #NLH01/082604a) lysates purchased from RDI Division of Fitzgerald Industries Intl (Concord, MA). All authors read and approved the final manuscript.

## Supplementary Material

Additional file 1 **HepG2_1795 unique proteins identified in the MudPIT mass spectra of HepG2 cells. **When Bioworks version 3.2 searched the HepG2 MudPIT data against NCBI nr database, 77831 hits were returned. Then, upon post-search processing and validation, only 1795 out of the original hits are confirmed. The post-search processing is based upon the criteria that: only peptides identified as possessing fully tryptic termini (containing up to two missed internal trypsin cleavage sites), with cross-correlation scores (X_corr_) greater than 1.9 for singly charged peptides, 2.3 for doubly charged peptides and 3.75 for triply charged peptides, are used for peptide identification. In addition, the delta-correlation scores (ΔC_n_) must be greater than 0.1 for peptide identification, and protein probability was set at ≤ 1e-002. Click here for file

Additional file 2 **Human Liver_1819 unique proteins identified in the MudPIT mass spectra of normal human liver tissue.** post-search criteria are identical to those of the HepG2 proteome of Additional File 1: Only 1819 validated, unique proteins are retained out of the 79961 original hits of the normal human liver proteome. Click here for file

Additional file 3 **MudPIT mass spectra of HepG2 cells (left) and normal human liver tissue (right).** There are twenty-four nano-LC/ESI-MS/MS spectra, two of which are 1D nano-LC/ESI-MS/MS. Twenty-two are 2D nano-LC/ESI-MS/MS spectra. Each MudPIT experiment consists of a 12-cycle run in which a 60-minute nano-LC/ESI-MS/MS gradient is run for each of: 1D, 2D, 2D (0 mM NH4COO), 2D (25 mM NH4COO), 2D (50 mM NH4COO), 2D (75 mM NH4COO), 2D (100 mM NH4COO), 2D (150 mM NH4COO), 2D (200 mM NH4COO), 2D (250 mM NH4COO), 2D (300 mM NH4COO) and 2D (500 mM NH4COO). Click here for file

Additional file 4 **HepG2_Sequence Table_BLASTP 2.2.13_ [Nov-27-2005].** The results of a Mapping operation are presented in the form of a Sequence Table, which consists of nine parameters (Headers): Sequence name, Seq description, Length, #hits, Maximum eValue, Similarity mean, number of Ontologies (GOs) found, the GO identification numbers of the found Ontologies, Enzyme (i.e. Enzyme Commission number). Click here for file

Additional file 5 **Human Liver_Sequence Table_BLASTP 2.2.13_ [Nov-27-2005].** Description of Sequence Table same as for Additional file 4. Click here for file

Additional file 6 **HepG2_Sequence Table_BLASTP 2.2.15_ [Oct-15-2006].** Description of Sequence Table same as for Additional file 4. Click here for file

Additional file 7 **Human Liver_Sequence Table_BLASTP 2.2.15_ [Oct-15-2006].** The results of a Mapping operation are presented in the form of a Sequence Table, which consists of nine parameters: Sequence name, Seq description, Length, #hits, Maximum eValue, Similarity mean, number of Ontologies (GOs) found, the GO identification numbers of the found Ontologies, Enzyme (i.e. Enzyme Commission number). Click here for file
