# Supplementary figures and images for: Dramatic down-regulation of oxidoreductases in human hepatocellular carcinoma hepG2 cells: proteomics and gene ontology unveiling new frontiers in cancer enzymology
Source: Proteome Sci. 2008 Oct 24;6:29. doi: 10.1186/1477-5956-6-29 (PMC2614416; doi:10.1186/1477-5956-6-29)

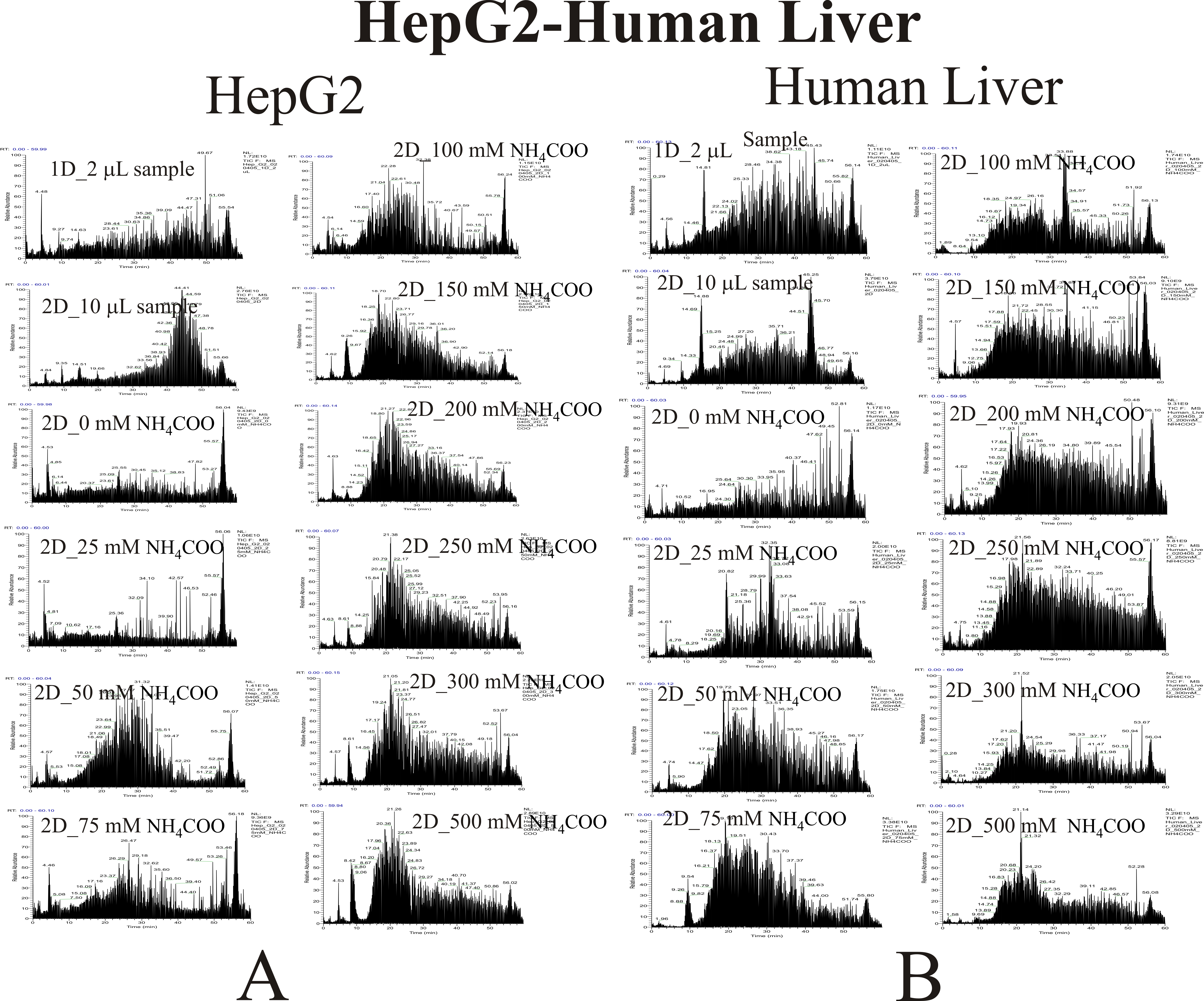

Supplement: Additional file 3 — MudPIT mass spectra of HepG2 cells (left) and normal human liver tissue (right). There are twenty-four nano-LC/ESI-MS/MS spectra, two of which are 1D nano-LC/ESI-MS/MS. Twenty-two are 2D nano-LC/ESI-MS/MS spectra. Each MudPIT experiment consists of a 12-cycle run in which a 60-minute nano-LC/ESI-MS/MS gradient is run for each of: 1D, 2D, 2D (0 mM NH4COO), 2D (25 mM NH4COO), 2D (50 mM NH4COO), 2D (75 mM NH4COO), 2D (100 mM NH4COO), 2D (150 mM NH4COO), 2D (200 mM NH4COO), 2D (250 mM NH4COO), 2D (300 mM NH4COO) and 2D (500 mM NH4COO). [file 1477-5956-6-29-S3.tiff]
